# Effects of NaHCO_3_ Stress on Black Locust (*Robinia pseudoacacia* L.) Physiology, Biochemistry, and Rhizosphere Bacterial Communities

**DOI:** 10.3390/microorganisms11122941

**Published:** 2023-12-08

**Authors:** Lulu Liu, Yu Chen, Liwen Zhang, Xueqi Bi, Fanjuan Meng, Qiuxiang Luo

**Affiliations:** Key Laboratory of Saline-Alkali Vegetation Ecology Restoration, College of Life Sciences, Ministry of Education, Northeast Forestry University, Harbin 150040, China; 13333362651@163.com (L.L.); 15556929593@163.com (Y.C.); zhangliwen0327@163.com (L.Z.); bxq1021@163.com (X.B.)

**Keywords:** rhizosphere bacteria, tetraploid black locust (*Robinia pseudoacacia* L.), NaHCO_3_-stress, community structure and diversity

## Abstract

Soil salinization has become an ecological and environmental problem that cannot be ignored. Tetraploid black locust (*Robinia pseudoacacia* L.) is a leguminous tree with characteristics of drought and saline-alkali tolerance. Rhizosphere bacteria are the primary functional microorganisms within the plant root system, and they play a crucial role in regulating plant growth and enhancing stress tolerance. However, there is still a lack of research on the effect of saline-alkali stress on the bacterial community structure in the rhizosphere of black locusts. In this study, we applied 0, 50, 100, and 150 mM NaHCO_3_ stress to diploid (2×) and tetraploid (4×) black locusts for 16 days. We used 16S rDNA sequencing to investigate the changes in the rhizosphere bacterial communities. Furthermore, we evaluated soil enzyme activity and plant physiological characteristics to explore the response of rhizosphere bacteria to NaHCO_3_ stress. The results demonstrated that the 4× plant exhibited superior alkali resistance compared to its 2× plant counterpart under NaHCO_3_ stress. Simultaneously, it was observed that low concentrations of NaHCO_3_ stress notably increased the abundance of rhizosphere bacteria in both plant types, while reducing their diversity. The impact of stress on the rhizosphere bacterial community weakened as the stress concentration increased. The application of NaHCO_3_ stress caused a significant change in the composition of the bacterial community in the rhizosphere. Additionally, alkaline salt stress influences the diversity of rhizosphere bacterial communities, which are linked to soil enzyme activities. These data will help us better understand the relationship between the dominant rhizosphere bacterial community and black locust. They will also provide a reference for further improving the alkali resistance of black locust by enhancing the soil bacterial community.

## 1. Introduction

Soil salinization has become one of the most significant soil problems that limit agricultural production. In light of shifting climate patterns, evolving land-use practices, and escalating population pressures, the threat of soil salinization poses a greater risk to agricultural output and ecosystems [1,2]. Regrettably, effective strategies to curb the increasing spread of saline soils remain elusive [3]. The consequences of soil salinization manifest through impediments such as stunted plant growth, premature withering, and a significant reduction in both the yield and quality of crops [4,5]. In saline-alkali soil, plants are subjected to the influences of ion toxicity, oxidative stress, and osmotic stress. These stress factors culminate in the diminished stability of plant cell membranes and cell wall structures, the inhibition of enzyme synthesis within plants, and a reduction in photosynthetic efficiency [6,7,8]. Extensive research has confirmed the impact of stress on crops such as soybeans [9], rice [10], and wheat [11] in saline-alkali soil environments.

Undoubtedly, saline soils present a challenging environment for habitation. However, within this inhospitable setting, microorganisms not only survive but also sustain diverse communities [12]. The rhizosphere is a crucial area for interactions among plants, the soil, and microbes. All microorganisms associated with the rhizosphere are collectively referred to as the rhizosphere microbiome (RM). The microbiome consists of beneficial microorganisms, such as mycorrhizal fungi and root-promoting bacteria (PGPR), as well as harmful microorganisms, such as soil-borne plant pathogens [13]. Under alkaline soil conditions, certain rhizosphere bacteria, such as *Bacillus* spp. from soybeans, have been shown to have the ability to promote plant growth [14]. The soil environment directly or indirectly affects the diversity of rhizosphere microorganisms and the community structure of microorganisms. Salinity stress can affect the community structure of rhizosphere soils in rice and maize. The relative abundance of bacteria, such as Proteobacteria and Firmicutes, increases with higher salinity [15]. This reciprocal relationship between plant stress and the composition of the rhizosphere microbial community, in turn, influences plant growth and development processes. A wealth of research highlights the role of rhizosphere bacteria in the plant stress response and in enhancing resistance to adverse conditions [16,17]. Beneficial rhizosphere microorganisms can enhance plant growth and development through various mechanisms. These mechanisms include facilitating plant nutrient uptake from the soil, modulating plant gene expression through the secretion of various organic compounds, and regulating plant physiological activities by secreting hormones. This enhances the plant’s stress resistance [18,19,20]. Actinobacteria sustain wheat growth under stress by promoting phytohormone production, thereby enhancing stress tolerance [21]. *Actinomyces* can promote the growth of soybeans and significantly improve the saline-alkali tolerance of soybeans [22]. Likewise, *Bacillus* sp. *strain SR-2-1* augments potato salt tolerance through the secretion of indoleacetic acid, which modulates ion uptake [23]. Rhizosphere bacteria play a crucial role in enhancing the resilience of plants to stress. Consequently, there exists a compelling need to further explore and study beneficial bacteria that provide resistance to saline-alkali stress.

Rhizosphere soil enzymes play a crucial role in the cycling of soil nutrients [24]. Assorted stress factors, including drought, salt stress, pests, and diseases, have the potential to alter the soil environment, which can subsequently affect soil enzyme activities [25]. These include enzymes that contribute to nutrient cycling and plant availability, such as urease, catalase, phosphatase, and sucrase. The composition and diversity of plant rhizosphere bacterial communities are influenced by soil physicochemical properties and soil enzyme activities [26]. Studying the relationship between soil enzyme activity and the microbial community structure can help us gain a better understanding of the functionality and stability of soil ecosystems.

Tetraploid black locust (*Robinia pseudoacacia* L.) was first identified in Korea through colchicine induction, which resulted in the production of seeds. This tetraploid variant exhibited significant advantages in terms of stress tolerance. Currently, research on the two varieties of black locusts has primarily focused on aspects such as resistance to neutral salt stress and drought stress [27,28]. Additionally, previous studies comparing the resistance of these two varieties have often emphasized differences in the expression of specific genes [29]. However, there are still relatively few studies in the field of rhizosphere bacteria.

In this study, we measured the physiological and biochemical indices, as well as the soil enzyme activities, of black locust. Additionally, we studied the bacterial community composition of the rhizosphere soil of diploid and tetraploid black locusts under alkali stress using high-throughput sequencing technology. The primary questions addressed in this study were as follows: (1) Do the characteristics of alkali stress and different ploidy affect the resistance of black locust by affecting the physiological characteristics of black locust and soil enzyme activity? (2) What changes have taken place in the rhizosphere bacterial community structure of black locust with different ploidy under alkali stress? (3) What are the possible factors that contribute to changes in the bacterial community in rhizosphere soil?

## 2. Materials and Methods

### 2.1. Plant Materials and Experimental Design

This study was conducted at the Key Laboratory of Saline-Alkali Vegetation Ecology Restoration, Ministry of Education, College of Life Science, Northeast Forestry University, Harbin. The examined two-year-old 2× and 4× black locust (*Robinia pseudoacacia* L.) seedlings were purchased from Hongsen High-Tech Forestry Co., Ltd. in Anhui City, China. We identified different ploidy black locust by flow cytometry [30]. The basic physical and chemical properties of the soil used in the experiment are shown in Table S1. The experiment commenced on 1 May 2022, with 2× and 4× black locust specimens of uniform morphological attributes, growth vigor, and size being transplanted into plastic nutrient pots measuring 20 cm × 30 cm. The soil weight in each pot was ensured to be 3 kg. One-fourth strength Hoagland nutrient solution was employed to ensure their survival and promote normal growth. After a 45-day acclimatization period, both the 2× and 4× black locusts were divided into four groups, each consisting of 9 pots per group, to undergo NaHCO_3_ stress. Tap water (pH 7.14 and EC 100 µS/cm) was employed as the control, with three different stress levels of gradient concentrations set at 50 mmol/L, 100 mmol/L, and 150 mmol/L of NaHCO_3_. According to the results of the pre-experiment, the potted soil requires approximately 2 L of solution for thorough irrigation. Therefore, 2 L of NaHCO_3_ solution with the appropriate concentration was used for each irrigation treatment. The same amount of stress solution was accurately measured with a measuring cylinder, and the stress solution was supplemented every two days. The control group was supplemented with an equal volume of water. A tray was placed under the nutrient bowl to prevent water and salt loss, thereby ensuring a consistent concentration of salt in the soil. Soil moisture sensors are used to monitor and control soil moisture levels, keeping them at 70% of field capacity. On the 16th day of the experiment, the growth parameters, such as plant height, stem diameter increment, and total biomass, were measured. Each treatment was conducted in triplicate. The measurement approach involved using the difference in values to compare the growth indicators on day 0 and day 16. The data obtained were then subjected to further analysis.

Mature and intact leaves located in the upper branches of the plant were collected for analysis. Three randomly selected plants were chosen as a sample for each treatment, and three repetitions were conducted. The leaves were promptly flash-frozen in liquid nitrogen. After sample collection, a portion of the leaves was retained to determine the relative conductivity and chlorophyll content. The remaining leaves were weighed and then frozen at −80 °C for subsequent assessment of various physiological and biochemical parameters. Soil samples were collected within 1 cm of the root base. Using a random five-point sampling approach, three plants were sampled for each treatment, and this process was repeated three times to obtain three replications. After sampling, the soil was sieved through a 60-mesh sieve and stored at 4 °C for analysis of soil pH and soil enzyme activity.

### 2.2. Determination of Plant Physiological Indexes and Soil Physical and Chemical Properties

Chlorophyll content was determined using colorimetry. The conductivity was measured using a conductivity meter. The Rayleigh DDS-307A conductivity meter was used for the measurement, and the obtained data will be used for further analysis. The concentration of malondialdehyde (MDA) was determined using the thiobarbituric acid method [31]. Superoxide dismutase (SOD) activity was determined using the nitroblue tetrazolium (NBT) method [32]. Catalase (CAT) activity was measured using ultraviolet absorption spectroscopy [33]. Peroxidase (POD) activity was determined using the guaiacol method [34]. The proline content (Pro) was determined using the acid ninhydrin method [35]. Soluble sugar (SS) content was determined using anthrone colorimetry [36]. Soluble protein (SP) content was determined using the Coomassie Brilliant Blue method [37]. Soil pH was measured using the Eutech CyberScan 1500, a high-precision benchtop pH meter. Soil urease (S-UE) activity was determined using the indophenol blue colorimetric method [38]. Soil sucrase (S-SC) activity was determined using the 3,5-dinitrosalicylic acid colorimetric method [39]. Soil alkaline phosphatase (S-AKP) activity was measured using the colorimetric method, with sodium phenylphosphate as the phosphorus source [40]. Soil catalase (S-CAT) activity was determined using the potassium permanganate titration method [41].

### 2.3. DNA Extraction, Bacterial 16S Gene Polymerase Chain Reaction Amplification and Sequencing

Total DNA was extracted from 0.5 g soil samples using a soil DNA extraction kit (OMEGA, Shanghai, China). DNA quantification was performed using Nanodrop, and the quality of DNA extraction was evaluated by 1.2% agarose gel electrophoresis. Subsequently, target sequences indicative of microbial community composition and diversity, such as microbial ribosomal RNA or specific gene fragments, were amplified using the bacterial 16S rRNA V3-V4 region primers 338F (5′-ACTCCTACGGGAGGCAGCA-3′) and 806R (5′-GGACTACHVGGGTWTCTAAT-3′) for PCR amplification. Sequencing libraries were prepared using the Illumina TruSeq Nano DNA LT Library Prep Kit. Following library preparation, the quality of the library was assessed using the Agilent High Sensitivity DNA Kit on the Agilent Bioanalyzer. Subsequently, paired-end sequencing was conducted using the MiSeq sequencing platform, and the corresponding sequencing reagent used was the MiSeq Reagent Kit V3 (600 cycles).

### 2.4. Statistical and Bioinformatics Analysis

All results are presented as the mean ± standard deviation (±SD) from three independent experiments. The data obtained from the experiments were subjected to statistical analysis and organized using Excel 2020 and SPSS 26.0 software. Duncan’s multiple comparison test was employed to assess differences among various concentration treatments, with distinct letter labels indicating significant differences between groups. Statistically significant differences in plant physiological indicators were observed among treatments (*p* < 0.05). A heatmap illustrating the changes in physiological indicators was generated using TBtools. Rank abundance curves were created using R scripting and the ggplot2 package. Rarefaction curves were constructed using QIIME2 (version 2019.4). Alpha diversity metrics, such as Chao1 and Observed species indices, were used to quantify community richness. Shannon and Simpson indices were used to assess community diversity, while Good’s coverage index was used to measure community coverage. These metrics were calculated using QIIME2 (2019.4). Box plots were then generated to visualize these metrics. Principal coordinate analysis (PCoA) based on Bray–Curtis dissimilarity was conducted using R to illustrate differences. Hierarchical clustering analysis was performed using the UPGMA algorithm and Bray–Curtis distance. This analysis was conducted using the uclust function from the R stats package (v4.1.3), resulting in the creation of a UPGMA clustering tree. Relative abundance heatmaps for bacterial phyla and genera were constructed using TBtools. LEfSe analysis was performed using the Galaxy online analysis platform. Redundancy analysis (RDA) graphs were generated using the ggplot2 and vegan packages in R (v4.1.3). 

## 3. Results

### 3.1. Physiological Responses of Plant under NaHCO_3_ Stress

**Figure 1:**
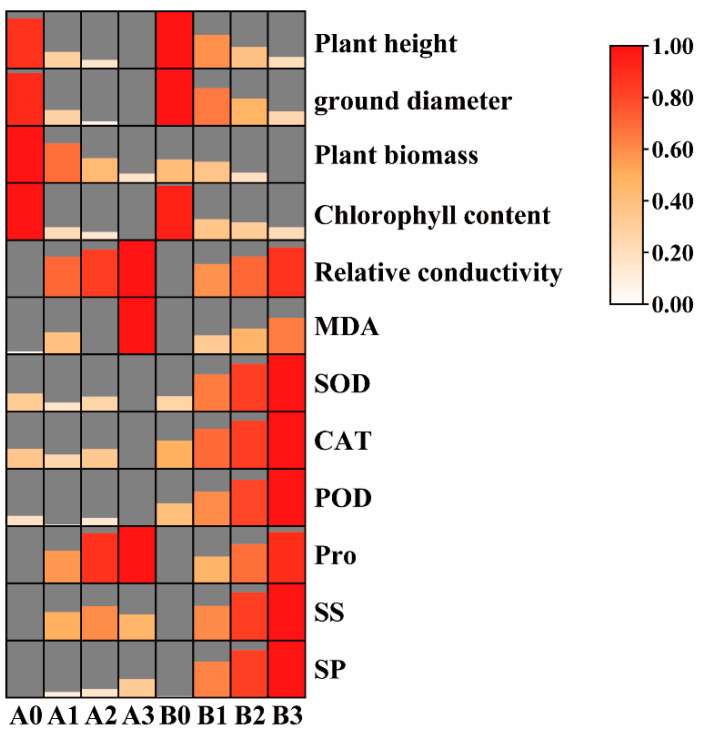
Heat map of plant physiological indices under NaHCO_3_ stress. X-axis: plants under stress treatments; Y-axis: plant physiological indicators. The color scale represents index changes. The value was the mean ± SD of 3 sample replicates. A0–A3: 2× plants treated with 0, 50, 100, and 150 mM NaHCO_3_. B0–B3: 4× plants treated with 0, 50, 100, and 150 mM NaHCO_3_. MAD (malondialdehyde), SOD (superoxide dismutase), POD (peroxidase), CAT (catalase), Pro (proline), SS (soluble sugar), SP (soluble protein).

Twelve growth-related, physiological, and biochemical indices were determined for 2× and 4× plants exposed to four concentration gradients of NaHCO_3_ stress treatment (Figure 1 and Table S2). Plant height, ground diameter, and total biomass, which are growth indicators, decreased significantly with increasing NaHCO_3_ stress concentrations in both the 2× and 4× treatment groups. This decline was reflected in the chlorophyll content of both the 2× and 4× plants. Conversely, metrics indicating membrane permeability, such as relative conductivity and MDA, showed an incremental increase in response to the stress. Moreover, the activities of antioxidant enzymes (SOD, POD, CAT) and osmoregulatory solutes (Pro, SS, SP) displayed an increasing pattern, significantly exceeding control levels in both the 2× and 4× treatment groups under NaHCO_3_-induced stress. Notably, when subjected to identical stress conditions, the 4× treatment group exhibited significantly higher values in terms of growth parameters, chlorophyll content, membrane permeability indices, antioxidant enzyme activities, and osmotic adjustment substances compared to the 2× treatment group.

### 3.2. Soil Enzyme Activities in Rhizosphere under NaHCO_3_ Stress

**Figure 2:**
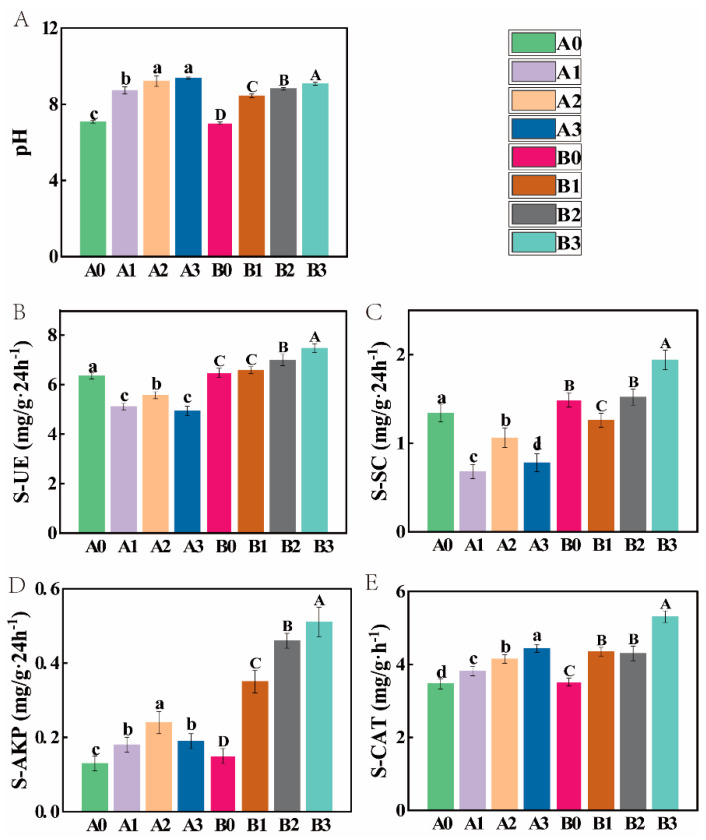
The pH (**A**), soil urease (S-UE) (**B**), soil sucrase (S-SC) (**C**), soil alkaline phosphatase (S-AKP) (**D**), and soil catalase (S-CAT) (**E**) of 2× and 4× plants under NaHCO_3_ stress. The value was the mean ± SD of 3 sample replicates. Lowercase or uppercase letters indicate significant differences in 2× or 4× plant stress gradients (*p* < 0.05, Tukey’s HSD post hoc analysis).

Under different concentrations of NaHCO_3_ stress, the pH of the rhizosphere soil of both 2× and 4× plants gradually increased with increasing stress concentrations (Figure 2). Under NaHCO_3_ stress, the activities of S-UE, S-SC, and S-AKP in the rhizosphere initially decreased, followed by an increase, and eventually declined for both plant species. S-CAT activity, on the other hand, showed a continuous increase. Remarkably, it is noteworthy that across all categories of soil enzyme activities, the 4× plants consistently exhibited significantly higher values than their 2× counterparts under equivalent stress conditions.

### 3.3. Responses of Rhizosphere Bacterial Diversity and Community Composition to NaHCO_3_ Stress

A total of 24 rhizosphere soil samples from plants were subjected to Illumina MiSeq/NovaSeq sequencing. Following primer removal, quality filtering, and clustering procedures, a final count of 47,301 operational taxonomic units (OTUs) was achieved. The rarefaction curve demonstrates the significant sequencing depth of the rhizosphere soil samples (Figure S1). The rank abundance curve displayed a gentle undulation, with a broad range along the horizontal axis, indicating a relatively high abundance and evenness of the rhizosphere soil samples (Figure S2).

**Figure 3:**
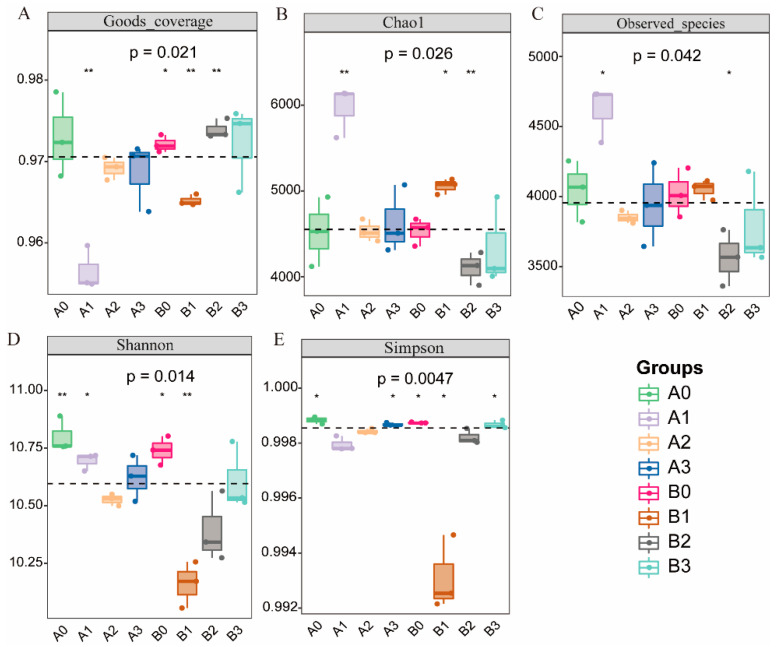
Alpha diversity of the rhizosphere bacterial community. (**A**) Good’s coverage index served as an indicator of coverage. (**B**) Chao1 and (**C**) observed species showed the species richness of the rhizosphere microbial community across different treatments. (**D**) Shannon, and (**E**) Simpson indices showed rhizosphere microbial community diversity among different treatments. The number under the diversity index label is the *p* value of the Kruskal−Wallis test. * *p* < 0.05; ** *p* < 0.01, Tukey’ test post hoc analysis.

As the concentration of NaHCO_3_ stress increased, the Good’s coverage indices of both 2× and 4× plant rhizosphere bacteria, as measured by Good’s coverage, were above 95%. This indicates a high level of coverage for the sequenced communities (Figure 3A). The Chao1 and observed species indices of rhizosphere bacteria for both ploidy levels demonstrated an increasing trend followed by a decrease, reaching the highest diversity in groups A1 and B1 (Figure 3B,C). Conversely, the Shannon and Simpson indices for both plant variants exhibited an initial decrease followed by an increase, reaching their lowest diversity in groups A1 and B1. (Figure 3D,E). Notably, in the context of 50 mM NaHCO_3_ stress, the Chao1, observed species, Shannon, and Simpson indices of the 4× plant were significantly lower than those of the 2× plant.

**Figure 4:**
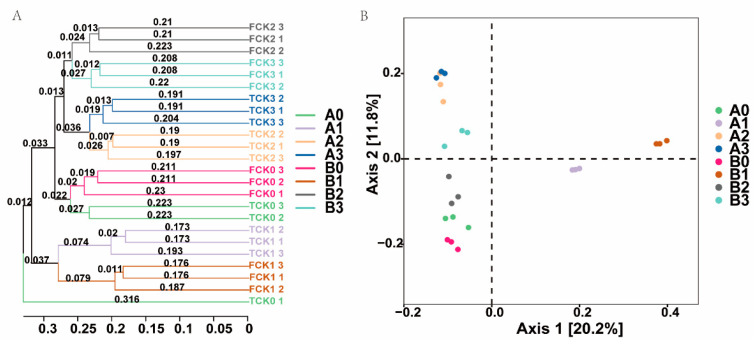
Cluster trees (**A**) and principal coordinates analysis (PCoA) (**B**) based on Bray–Curtis distance metrics.

Utilizing the Bray–Curtis based clustering tree (Figure 4A) analysis, it becomes evident that under the influence of identical NaHCO_3_ stress, the 2× and 4× plants exhibit a notable degree of similarity. Notably, groups A1 and B1 exhibit heightened inter-group variability in comparison to other groups. Conversely, groups B2 and B3 showcase stronger similarity to groups A0 and B0. Using the Bray–Curtis based principal coordinates analysis (PCoA) (Figure 4B), the initial two principal components (Axis 1 and Axis 2) of the PCoA explain 31.6% and 23.4% of the total variance. The results highlight that the application of 50 mM NaHCO_3_ stress causes noticeable changes in the bacterial community composition in both the 2× and 4× plants, relative to both the control and stress groups. However, it is noteworthy that under identical stress conditions, there is no statistically significant disparity in the rhizosphere bacteria community structure between the two plant variants.

**Figure 5:**
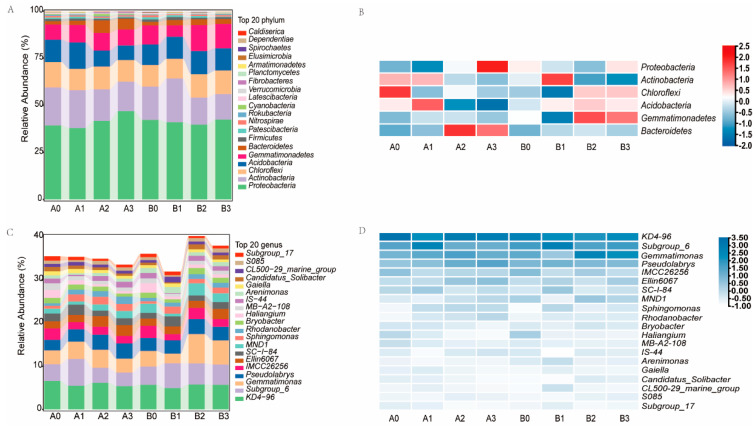
Analysis of rhizosphere bacterial community composition under different treatments. The top 20 phyla in abundance of rhizosphere bacteria from 2× and 4× plants under different NaHCO_3_ stress (**A**), phyla with an abundance greater than 1% (**B**), and the top 20 genera in abundance (**C**,**D**).

In the rhizosphere soil of both 2× and 4× plants, six bacterial phyla were identified, each with an average abundance exceeding 1%. Notably, the most abundant phylum was Proteobacteria, followed by Actinobacteria, Chloroflexi, Acidobacteria, Gemmatimonadetes, and Bacteroidetes (Figure 5A,B). Under NaHCO_3_ stress, the abundance of Proteobacteria, Acidobacteria, and Bacteroidetes significantly increased in the 2× plant. Similarly, under NaHCO_3_ stress, the abundance of Actinobacteria and Gemmatimonadetes significantly increased in the 4× plant. Importantly, it is noteworthy that post NaHCO_3_ stress, the abundance of Proteobacteria, Actinobacteria, and Bacteroidetes in the rhizosphere of the 4× plant was significantly lower than in the 2× plant. However, the abundance of Acidobacteria and Gemmatimonadetes was significantly higher in the 4× plant compared to the 2× plant.

The top 20 genera in the total abundance of rhizosphere bacteria at the genus level of 2× and 4× plants are shown in the figure (Figure 5C,D). Under NaHCO_3_ stress, in the genus-level rhizosphere bacteria of the 2× plant, there was a marked increase in the abundance of *Subgroup_6*, *Gemmatimonas*, *Pseudolabrys*, *Ellin6067*, *MND1*, *Sphingomonas*, *Rhodanobacter*, *IS−44*, *CL500_29_marine_group*, and *S085*. Likewise, in response to NaHCO_3_ stress, the abundance of *Subgroup_6*, *Gemmatimonas*, *Ellin6067*, *SC−I−84*, *MND1*, *Sphingomonas*, *Bryobacter*, *Arenimonas*, *Gaiella*, *Candidatus_Solibacter*, *CL500_29_marine_group*, S580, and *Subgroup_17* exhibited a notable increase in the rhizosphere bacteria of the 4× plant. It’s noteworthy that after NaHCO_3_ stress, in the rhizosphere of the 4× plant, the abundance of *Gemmatimonas*, *MND1*, *Bryobacter*, *Arenimonas*, *Candidatus_Solibacter*, and *CL500_29_marine_group* was significantly higher compared to the 2× plant.

**Figure 6:**
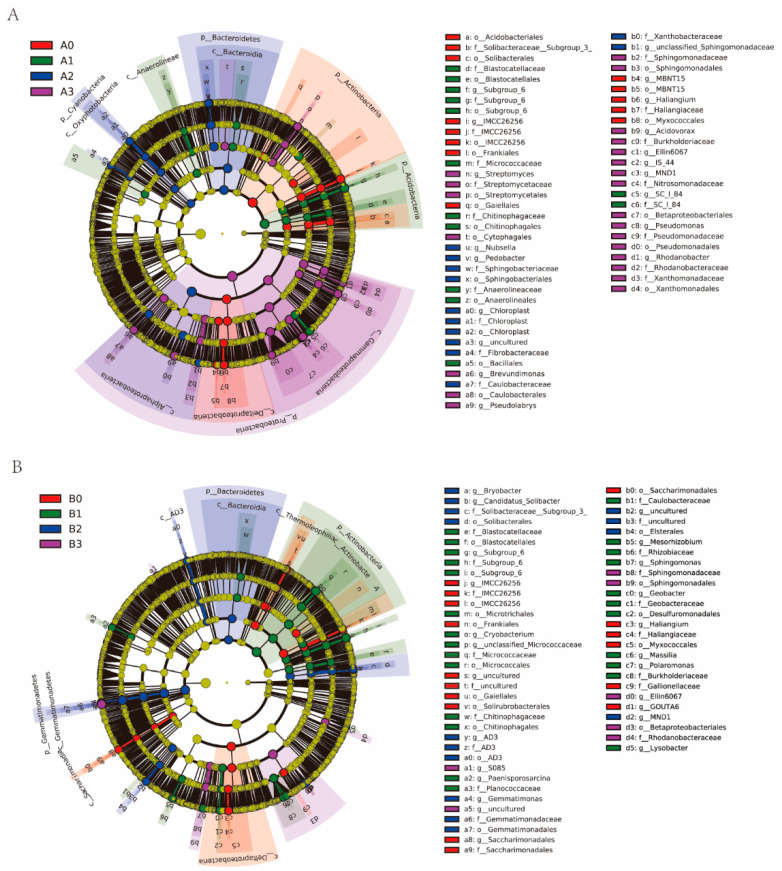
Linear discriminant analysis effect size (LEfSe) analysis was conducted to assess the bacterial abundance from phylum to genus (LDA threshold score ≥ 3.5) in the rhizosphere microbial communities of 2× plants (**A**) and 4× plants (**B**) under different stress concentrations.

Utilizing linear discriminant analysis (LDA) effect size analysis (LEfSe), we compared the bacterial composition at the phylum to genus levels (from phyla to genera) between 2× and 4× plants under different NaHCO_3_ treatments (Figure 6 and Figure S3). In the A1 group, the enriched bacteria at the genus level included *Subgroup_6* and *SC−1−84*. The A2 group exhibited an enrichment of genus-level bacteria such as *Acidovorax*, *Nubsella*, *Chloroplast*, and *Pedobacter*. Similarly, the A3 group displayed an enrichment of bacteria at the genus level, including *Pseudomonas*, *Rhodanobacter*, *Brevundimonas*, *Pseudolabrys*, *IS-44*, *MND1*, *Ellin6067*, and *Acidovorax*. Biomarkers for the 2× plant were primarily found in the phyla Proteobacteria, Actinobacteria, Bacteroidetes, and Cyanobacteria. Within the B1 group, the enriched bacteria at the genus level consisted of *Cryobacterium*, *Subgroup 6*, *Massilia*, *Sphingomonas*, *Mesorhizobium*, *Lysobacter*, *Paenisporosarcina*, and *Polaromonas*. The B2 group exhibited an enrichment of bacteria at the genus level, including *Gemmatimonas*, *MND1*, *Candidatus_Solibacter*, and *Bryobacter*. In the B3 group, the enriched bacteria at the genus level included *Ellin6067* and *S085*. Biomarkers for the 4× plant were primarily found within the phyla Gemmatimonadetes, Acidobacteriia, and Bacteroidetes.

### 3.4. Relationship between Soil Enzymes and the Rhizosphere Bacterial Community

**Figure 7:**
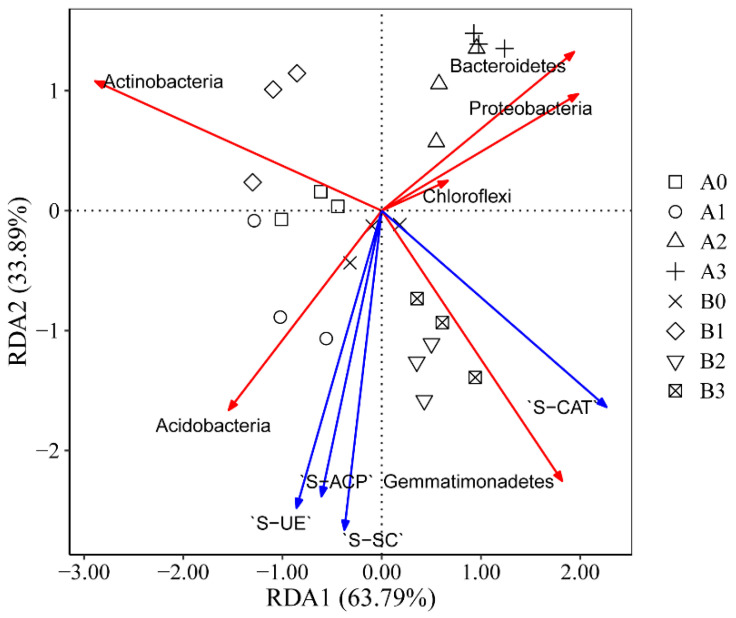
Redundancy analysis (RDA) of rhizosphere bacterial relative abundance at the phylum level and soil enzymes. The blue arrow represents soil enzyme activity, and the red arrow represents the top 6 rhizosphere bacteria at the phylum level.

The RDA analysis depicted reveals the correlation between bacterial communities at the rhizosphere level and rhizosphere soil enzymes (Figure 7 and Table S3). The first ordination axis, RDA1, and the second ordination axis, RDA2, explain 63.79% and 33.89% of the variance in the bacterial communities, respectively. The outcomes demonstrate a positive correlation between soil enzymes S-AKP, S-SC, and S-UE with the phylum Acidobacteria. Additionally, a positive correlation is observed between S-CAT and the phylum Gemmatimonadetes.

## 4. Discussion

### 4.1. Physiological Responses of Plant to NaHCO_3_ Stress

NaHCO_3_ stress directly impacts the normal physiological activities of plants, leading to detrimental effects on plant growth [42]. Our investigation has revealed that, under equivalent NaHCO_3_ concentrations, the growth indicators, antioxidant enzyme activities, and osmotic regulatory substance levels of the 4× plant are significantly higher than those in the 2× plant. Moreover, the membrane permeability index of the 4× plant is significantly higher than that of the 2× plant (Figure 1 and Table S1). These results are similar to previous studies on the salt resistance of tetraploid black locust [43,44], indicating that the 4× plant is more alkali-resistant than the 2× plant. It has been suggested that there may be a correlation between increased soil enzyme activity and increased plant stress tolerance [45]. Under NaHCO_3_ stress, significant changes in soil enzyme activity were observed in both plant species. Remarkably, various soil enzyme activities of the 4× plant were significantly higher than those of the 2× plant (Figure 2). This disparity may be correlated with the increased tolerance of the 4× plant to alkali stress. However, this conclusion merely scratches the surface, as plant responses to stress are influenced by multiple factors. Consequently, further comprehensive exploration remains imperative in future research endeavors.

### 4.2. The Alkali Tolerance of a Plant Is Related to the Composition of the Bacterial Community

Rhizobacteria play a crucial role in plant growth and metabolism. Under saline-alkali stress, the growth of rhizosphere microorganisms was inhibited and the bacterial community was changed [46,47]. This study utilizes 16S rRNA amplicon sequencing technology to investigate the rhizobacterial community structure of plants under NaHCO_3_ stress. The research findings demonstrate that under high-concentration NaHCO_3_ stress, the diversity changes in the rhizosphere communities for both types of plants were not conspicuous. However, after being exposed to 50 mM NaHCO_3_ stress, significant alterations were observed in the alpha-diversity of bacterial communities in both plant species (Figure 3). Notably, the abundance and diversity of rhizosphere bacteria in the 4× plant were considerably lower than those in the 2× plant. These results indicate that both the plant species and the distinct concentrations of NaHCO_3_ stress exert a certain influence on the α-diversity of rhizosphere bacteria. Furthermore, it appears that under NaHCO_3_ stress, the bacterial community structure is more stable in the 4× plant. Nonetheless, further research is required to validate these hypotheses. We conducted comparisons at both the phylum and genus levels to determine the composition of major bacterial taxa in the rhizosphere of two plant species. Our goal was to understand the differences in the composition of rhizosphere bacterial communities under different stress conditions. Experimental findings reveal that there is no significant difference in the composition of rhizosphere bacterial communities at the phylum level between the two plant species. However, Actinobacteria, Chloroflexi, and Gemmatimonadetes exhibit an increasing trend in abundance in the rhizosphere soil of the 4× plant, while demonstrating a decreasing trend in the rhizosphere soil of the 2× plant. Moreover, the abundances of Actinobacteria and Gemmatimonadetes are significantly higher in the rhizosphere of the 4× plant compared to the 2× plant (Figure 5A,B). Previous studies have shown that these bacterial phyla are responsive to various environmental stresses, such as salt [48], drought [49], and heavy metal stress [50].

Further comparing the composition of rhizosphere bacteria at the genus level, it was found that the following genera had significantly higher bacterial abundance in the rhizosphere of the 4× plant compared to the 2× plant after NaHCO_3_ stress: *Gemmatimonas*, *MND1*, *Bryobacter*, *Arenimonas*, *Candidatus_Solibacter*, *and CL500_29_marine_group*. These genera belong to Gemmatimonadetes, Proteobacteria, Actinobacteria, and Acidobacteria, respectively (Figure 5C,D). Pseudolabrys and Actinobacteria have been shown to contribute to nitrogen fixation in soils, thereby improving nitrogen utilization efficiency and promoting biomass accumulation [51,52]. Certain members of Actinobacteria have shown resistance to alkaline conditions, high temperatures, and drought [53]. LEfSe analysis showed that the rhizosphere bacterial genera enriched in the 4× plant stress group were mainly *Cryobacterium*, *Subgroup_6*, *Massilia*, *Sphingomonas*, *Mesorhizobium*, *Lysobacter*, *Paenisporosarcina*, *Polaromonas*, *Gemmatimonas*, *MND1*, *Candidatus_Solibacter*, *Bryobacter*, *Ellin6067*, and *S085*. Most of these plant rhizosphere communities belong to Gemmatimonadetes, Acidobacteriia, and Bacteroidetes (Figure 6B). *Sphingomonas* is known for its role in detoxifying reactive oxygen species produced by plants in the rhizosphere [54]. *Mesorhizobium* displays a degree of tolerance to temperature, salt, and pH stress [55]. *Gemmatimonas* has been associated with enhanced plant tolerance to cadmium toxicity [56]. *MND1* plays a crucial role in the mineralization of both organic and inorganic phosphorus, which facilitates plant phosphorus absorption [57]. *Candidatus_Solibacter* is capable of decomposing organic matter and utilizing carbon sources, and its abundance is positively correlated with soil organic matter content [58]. *Ellin6067*, a nitrate-reducing bacterium, converts inorganic nitrogen into nitrate, which is a nutrient that can be directly absorbed by plants [59]. In summary, these rhizosphere bacteria that are enriched under stress promote plant growth and development through different mechanisms of action. It is speculated that the significant increase in abundance may be attributed to the stronger alkaline resistance of the 4× plant compared to the 2× plant. However, further in-depth and detailed studies are needed to understand the underlying reasons.

### 4.3. Interactions between Soil Enzymes and Plant Rhizosphere Bacteria under NaHCO_3_ Stress

Soil enzymes primarily originate from soil microorganisms and plant root systems [60]. Notably, enzymes such as S-UE, S-SC, S-AKP, and S-CAT play pivotal roles in soil carbon, nitrogen, and phosphorus cycling [61,62]. Rhizosphere bacteria facilitate the degradation of organic matter, promoting the cycling of C, N, and P, thereby influencing plant growth [63,64]. In our experiment, the RDA analysis revealed positive correlations between S-AKP, S-SC, S-UE, and the Acidobacteria. Simultaneously, a positive correlation was observed between S-CAT and the Gemmatimonadetes (Figure 7).

Studies indicate that S-UE plays a crucial role in the nitrogen cycling process in soil by facilitating the breakdown of urea. This process aids in the release and recycling of nitrogen [65]. The activity of the S-SC is closely associated with the cycling of soil organic carbon and microbial metabolism. Through the process of sucrose degradation, microorganisms are able to obtain energy and carbon sources, which in turn sustain their growth and metabolism [66]. S-AKP, as a crucial element in rhizosphere secretion, plays a key role in soil nutrient metabolism by participating in the mineralization of rhizosphere soil phosphorus and the transformation of soil protein components [67]. Furthermore, within the phylum Acidobacteria, certain bacterial members are known to participate in processes such as carbohydrate degradation, demonstrating their relevance to carbon cycling [68,69]. Acidobacteria also possess genes for catalyzing the metabolism of inorganic and organic nitrogen sources. They efficiently reduce nitrates, nitrites, and possibly nitric oxide, indicating their active participation in the nitrogen cycle [70]. S-CAT is widely present in soil and functions to decompose harmful hydrogen peroxide in plants. It has a certain relationship with the conversion of soil carbon and the organic matter content [71]. S-CAT may be involved in nitrogen metabolism, specifically in the formation and breakdown of nitrites and nitrates in the soil. Nitrogen plays a crucial role in the cycling and transformation of nitrogen in the soil [72]. Gemmatimonadetes have also been found to participate in carbon cycling processes in the soil, including the decomposition of organic matter and carbon fixation. This affects the balance of soil organic matter and the stability of carbon cycling [73,74]. The bacteria within the Gemmatimonadetes phylum contribute to the reduction of N_2_O in soil, actively participating in the cycling of soil nitrogen [75]. This explains the strong correlation revealed by RDA analysis between specific rhizosphere bacteria and soil enzyme characteristics. Therefore, we speculate that the interactions between soil enzymes and rhizosphere bacteria may contribute to promoting the cycling of C and nitrogen N in soil nutrients. This, in turn, ensures an ample supply of essential nutrients to plant roots, especially under NaHCO_3_ stress. Certainly, the underlying mechanisms of these interactions are undoubtedly complex. Further research is necessary to comprehensively and thoroughly explore these intricate relationships.

## 5. Conclusions

This study sheds light on the resistance of black locust to alkaline salt stress, attributing it to both physiological adjustments and regulation by rhizosphere bacteria. The maintenance of higher physiological and biochemical indices during NaHCO_3_ stress contributes to the superior tolerance to alkaline stress compared to the 2× plant. The diversity of rhizosphere soil bacterial communities is altered by NaHCO_3_ stress in both plant species. Moreover, NaHCO_3_ stress modifies the composition of rhizosphere bacterial communities in black locust. Rhizosphere bacteria enriched under NaHCO_3_ stress may play a crucial role in the microbial-mediated mechanism of the 4× plant tolerance to alkaline salt stress. This study also highlights that when exposed to alkaline salt stress, soil enzymes exhibit a strong correlation with several major bacterial communities in black locust. This suggests that soil enzymes may play a significant role in shaping the structure of bacterial communities. This study offers a novel perspective on the resistance of black locust to saline-alkali stress. This also provides a theoretical basis for the future utilization of tetraploid black locust to improve methods for restoring saline-alkaline land.

## Data Availability

Data are contained within the article and supplementary materials.

## Supplementary Materials

The following supporting information can be downloaded at: https://www.mdpi.com/article/10.3390/microorganisms11122941/s1, Figure S1: Rank abundance curves were generated using bacterial 16S rRNA gene sequences obtained from amplicon sequencing; Figure S2: Rarefaction curve of bacterial 16S rDNA gene sequences obtained from amplicon sequencing; Figure S3. Linear discriminant analysis effect size (LEfSe) analysis was conducted to assess the bacterial abundance from phylum to genus (LDA threshold score ≥ 3.5) in the rhizosphere microbial communities of 2× plants (A) and 4× plants (B) under different stress concentrations; Table S1. Basic properties of test soil; Table S2: Physiological responses of plants under NaHCO_3_ stress; Table S3: The correlation between each factor and the principal component of the RDA plot.
