# Supplementary material for: Effects of NaHCO3 Stress on Black Locust (Robinia pseudoacacia L.) Physiology, Biochemistry, and Rhizosphere Bacterial Communities
Source: Microorganisms. 2023 Dec 8;11(12):2941. doi: 10.3390/microorganisms11122941 (PMC10745695; doi:10.3390/microorganisms11122941)
Supplement: Supplementary file 1 [file microorganisms-11-02941-s001.zip › microorganisms-2730700-supplementary.pdf]

Table S1. Basic properties of test soil.

physicochemical indexes	numerical value
pH	6.6
organic matter content	20.36 g/kg
total nitrogen content	99.8 g/kg
total phosphorus content	22.4 g/kg
total potassium content	7.11 g/kg
alkaline hydrolyzed nitrogen	136 mg/kg
available phosphorus	83 mg/kg
available potassium	77.1 mg/kg

Table S2. Physiological responses of plants under $NaHCO_3$ stress.

Variety	Treatment	Plant height (cm)	ground diameter (mm)	Plant biomass (g)	Chlorophyll content (mg/g)	Relative conductivity (%)	MDA (μmol/g)	SOD (U/g)	POD(U/g·min^{-1})	CAT (U/g·min^{-1})	Pro(μg/g)	SS(mg/g)	SP(mg/g)
2×	A0	11.47±1.02a	1.04±0.09a	17.43±0.26a	25.27±0.56a	22.74±2.08d	3.46±0.36d	10.91±0.5a	15.87±1.4a	37.48±1.11a	21.54±1.18d	27.25±1.03c	6.15±0.39c
	A1	6.31±0.59b	0.50±0.02b	16±0.48b	11.22±0.5b	44.55±0.88c	7.85±0.3c	9.08±0.19b	13.2±0.58b	34.48±0.84b	134.61±3.6c	43.25±1.48b	6.79±0.25b
	A2	5.08±0.52bc	0.33±0.04c	14.82±0.32c	9.76±0.48c	48.57±0.64b	2.77±0.54b	10.29±0.47a	15.18±0.71a	37.19±0.66a	196.61±4.04b	46.6±1.06a	7.12±0.28b
	A3	3.76±0.38c	0.27±0.03c	13.58±0.33d	7.19±0.22d	53.72±1.05a	15.87±0.53a	7.3±0.38c	12.76±0.61b	27.28±0.46b	221.51±4.55a	41.91±1.32b	8.25±0.32a
4×	B0	12.57±0.62A	1.11±0.12A	14.73±0.38A	24.23±0.58A	22.27±1.62D	3±0.26D	10.36±0.54D	19.72±0.88D	41.53±1.07D	22.61±2.15D	27.44±1.05D	6.32±0.41D
	B1	8.91±0.26bB	0.82±0.05B	14.53±0.3A	13.77±0.71B	40.59±0.82B	7.11±0.28C	14.69±0.53C	23.25±0.54C	47.42±0.81C	114.68±2.96C	46.78±0.87C	10.23±0.4C
	B2	7.06±0.35C	0.67±0.02C	13.66±0.14B	12.83±0.43B	44.6±0.83B	8.71±0.55B	16.82±0.56B	26.9±0.71B	51.49±0.95B	158.31±4.19B	54.35±1.77B	11.55±0.33B
	B3	5.51±0.23D	0.48±0.02D	12.84±0.18C	11.22±0.54C	49.66±0.66A	11.2±0.47A	18.68±0.57A	30.21±0.64A	56.14±0.66A	200.6±5.64A	59.57±2.09A	12.65±0.31A
ANOVA	Variety	p<0.001	p<0.001	p<0.001	p<0.001	p<0.001	p<0.001	p<0.001	p<0.001	p<0.001	p<0.001	p<0.001	p<0.001
	Treatment	p<0.001	p<0.001	p<0.001	p<0.001	p<0.001	p<0.001	p<0.001	p<0.001	p<0.001	p<0.001	p<0.001	p<0.001
	Variety*Treatment	P=0.319	P=0.026	P=0.003	p<0.001	P=0.044	p<0.001	p<0.001	p<0.001	p<0.001	p<0.001	p<0.001	p<0.001

The values represent the mean of three soil samples. The Tukey's test showed that the same column of different lowercase letters indicated a significant difference in the 2× plants' $NAHCO_3$ stress treatment, while different uppercase letters indicated a significant difference in the 4× plants' $NaHCO_3$ stress treatment ($P < 0.05$). Significant at the 0.001 level. A0, A1, A2, and A3 represent 2× plants subjected to 0, 50, 100, and 150 mM $NaHCO_3$ stress treatment, B0, B1, B2, and B3 represent 4× plants subjected to 0, 50, 100, and 150 mM $NaHCO_3$ stress treatment.

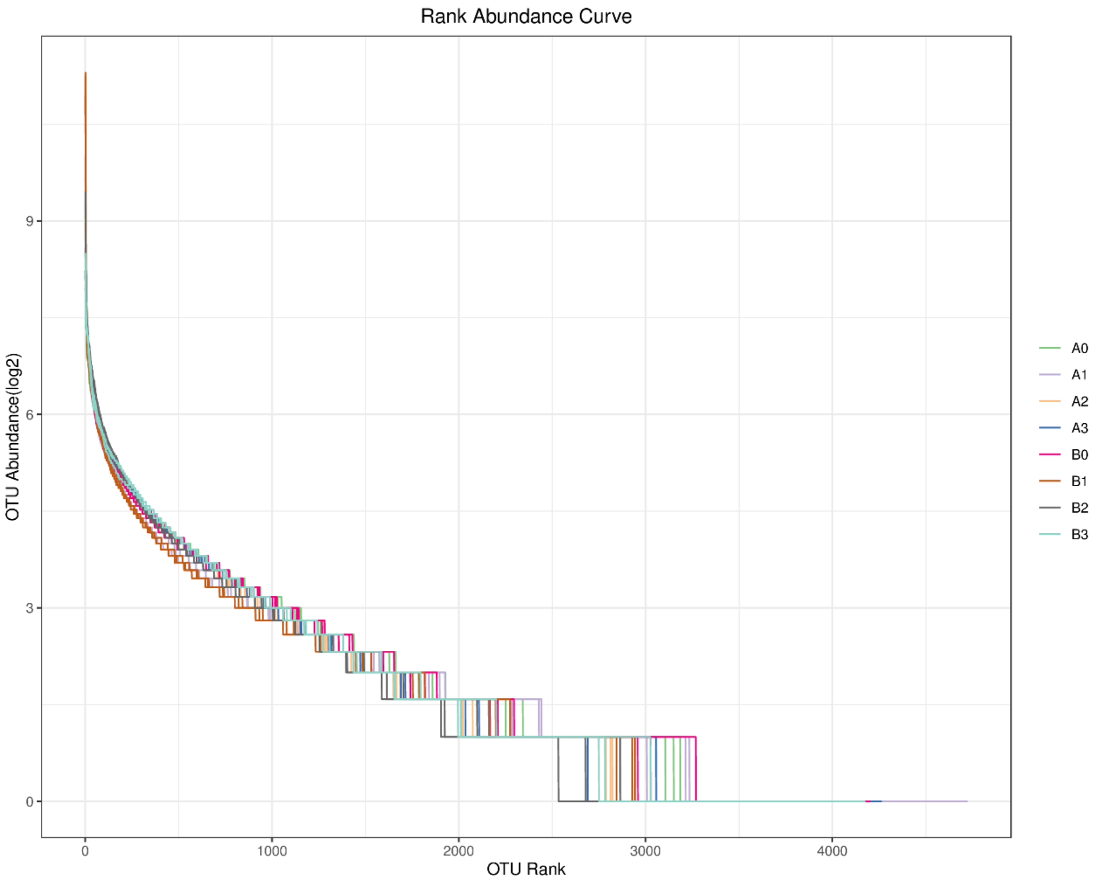

Figure S1. Rank abundance curves were generated using bacterial 16S rRNA gene sequences obtained from amplicon sequencing.

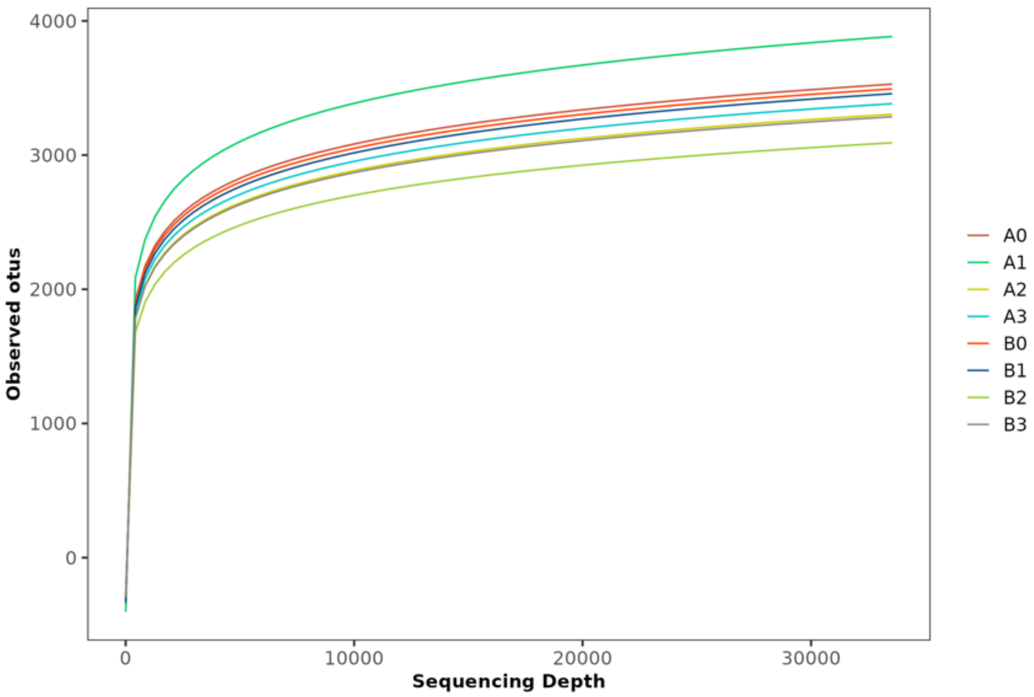

Figure S2. Rarefaction Curve of bacterial 16S rRNA gene sequences obtained from amplicon sequencing.

Table S3. The correlation between each factor and the principal component of the RDA plot is displayed.

Indicators	R2	Pr>(r)
S-UE	0.3911	0.008**
S-ACP	0.3424	0.014*
S-SC	0.4129	0.005**
S-CAT	0.6646	0.001**

The R2 of the environmental factor of the RDA chart and the significance p-value.

*P value < 0.05； **P value < 0.01; ***P value < 0.001.

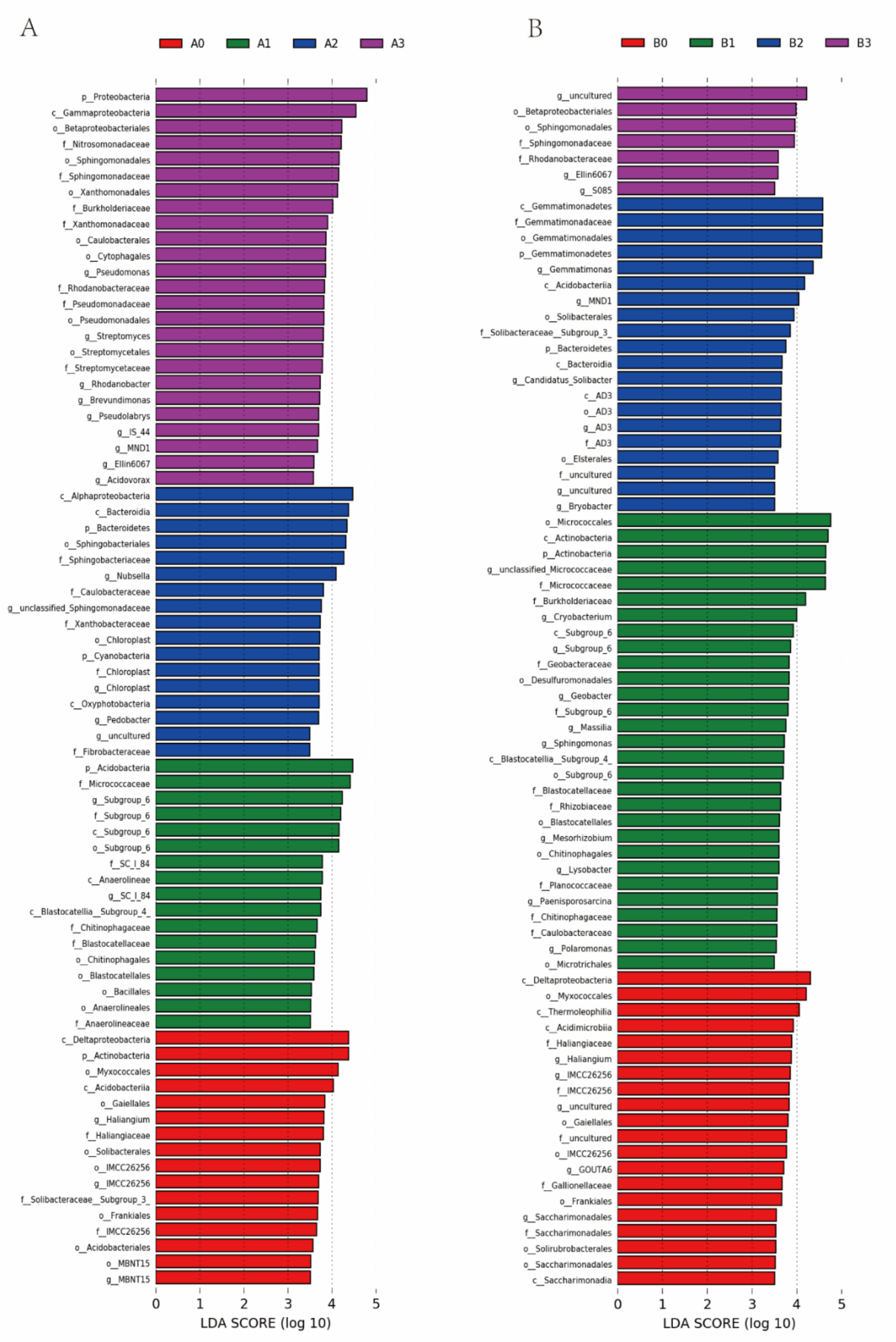

Figure S3. Linear discriminant analysis effect size (LEfSe) analysis was conducted to assess the bacterial abundance from phylum to genus (LDA threshold score ≥ 3.5) in the rhizosphere microbial communities of 2 × plant (A) and 4 × plant (B) under different stress concentrations.
